# Genetic Polymorphisms of the *CASP8* Gene Promoter May Not Be Associated with Colorectal Cancer in Han Chinese from Southwest China

**DOI:** 10.1371/journal.pone.0067577

**Published:** 2013-07-02

**Authors:** Mei-Sheng Xiao, Le Chang, Wen-Liang Li, Yong-Sheng Du, Yue Pan, Deng-Feng Zhang, Yu Wen, Juan Luo, Xiao-Yan Li, Yong-Gang Yao

**Affiliations:** 1 Key Laboratory of Animal Models and Human Disease Mechanisms of the Chinese Academy of Sciences & Yunnan Province, Kunming Institute of Zoology, Kunming, China; 2 Department of Gastroenterology, The First Affiliated Hospital of Kunming Medical University, Kunming, China; 3 Department of Oncology, The First Affiliated Hospital of Kunming Medical University, Kunming, China; 4 University of Chinese Academy of Sciences, Beijing, China; Vanderbilt University, United States of America

## Abstract

**Purpose:**

Caspase 8 (CASP8) plays a critical role in the apoptotic pathway and aberrant regulation of this pathway causes many diseases including cancers. Genetic variants rs3834129 (CTTACT/−) and rs3769821 (T/C) in the promoter region of the *CASP8* gene were documented to be associated with multiple solid cancers and non-Hodgkin’s lymphoma (NHL), respectively, despite of some controversies. We aimed to discern potential association of these two variants and rs113686495 (CTGTCATT/−), as well as *CASP8* mRNA and protein expression levels with colorectal cancer (CRC) in Han Chinese.

**Methods:**

We genotyped *CASP8* genetic variants in 305 CRC patients and 342 healthy individuals from Kunming, Southwest China. Expression levels of *CASP8* mRNA and protein were quantified in paired cancerous and paracancerous normal tissues by using real-time quantitative PCR and western blot, respectively. We compared the frequencies of alleles, genotypes, and haplotypes between the cases and controls. Correlation of *CASP8* mRNA and protein expression levels in paired cancerous and paracancerous normal tissues from patients with different genotypes and clinical expression were also evaluated.

**Results:**

There was no association of the *CASP8* genetic variants with CRC in our case-control study. The *CASP8* gene mRNA expression levels in cancerous and paracancerous normal tissues were similar and there was no significant difference between subjects with different genotypes and clinical features. However, we found that CASP8 protein level was significantly lower in cancerous tissues than in paired paracancerous normal tissues.

**Conclusions:**

Our results suggest that the three *CASP8* genetic variants may not be associated with CRC risk in Han Chinese from southwest China. Aberrant CASP8 protein expression may play a role in the pathogenesis of CRC.

## Introduction

Colorectal cancer (CRC) is one of the most prevalent cancers around the world, with a 5-year survival rate of 30–65% [1]. Although the majority of CRC (nearly 80%) is sporadic [2], [3], about 35% of CRC patients can be attributed to genetic background according to the study of monozygotic twins [3], implying that both genetic and environmental factors have pivotal roles in the pathogenesis of CRC. Many people were exposed to environmental risk factors, such as smoking [4], drinking [4], unhealthy dietary and lifestyles [5], [6], but only some of them suffered from CRC, suggesting that genetic variations partially determine the susceptibility to CRC.

Apoptosis, also called programmed cell death, is involved in maintaining tissue homeostasis, development and eliminating unwanted cells in multicellular organisms [7]. Dysfunction of this process would result in tumorigenesis [8]. Apoptotic cell death is mediated by a family of highly conserved caspases (cystein-dependent aspartate-specific proteases), which can be divided into “initiator” caspases and “effector” caspases [9]. There are mainly two apoptotic pathways in human: the extrinsic or receptor-mediated pathway and the intrinsic or mitochondrial pathway, both employ caspases cascade [10], [11], [12].

Caspase 8, encoded by the *CASP8* gene (which is located on chromosome 2q33-q34 and has 14 exons), functions as an initiator caspase in the apoptotic pathway and a crucial defensive barrier against malignant proliferation and tumorigenesis [7], [8], [11], [13]. The indel polymorphism, rs3834129 (CTTACT/−, written as 6 bp/del in the following text), in the promoter region of the *CASP8* gene was reported to be associated with susceptibility to a wide range of cancers including CRC in Chinese populations [14]. Although this variant was subsequently reported to be associated with the risk of coal workers and bladder cancers in Chinese populations [15], [16], the positive association was not replicated in subsequent large scale case-control studies in European and American populations [17], [18]. Genotypes TC and CC of another single nucleotide polymorphism (SNP), rs3769821 (T/C), which is also located in the promoter region of the *CASP8* gene, was found to influence genetic susceptibility to non-Hodgkin’s lymphoma (NHL) in a pooled analysis of three populations from the United States of America and Australia [19]. However, this positive result was not confirmed in our recent genetic analysis for Han Chinese with NHL and luciferase assay [20].

To discern whether rs3834129 and rs3769821 contribute to genetic susceptibility to CRC in Han Chinese from southwest China, we genotyped these two variants, together with rs113686495 (CTGTCATT/−, written as 8 bp/del in the following text; which is located at 50 bp downstream of rs3769821). We further quantified mRNA expression level of the *CASP8* gene in both cancerous and paracancerous normal tissues of CRC patients with different genotypes to identify potential effect of different alleles on gene expression. In addition, we compared mRNA levels in CRC patients with different clinical characteristics. The CASP8 protein level was measured in paired cancerous and paracancerous normal tissues from a total of 39 patients, to compare with the pattern of mRNA expression. Our results showed that *CASP8* genetic variants and mRNA expression level were unlikely to be associated with CRC in Han Chinese. However, we found that cancerous tissues had a significantly lower level of CASP8 protein than paracancerous normal tissues, suggesting that potential post-transcriptional regulation of this gene plays an active role in the development of CRC.

## Materials and Methods

### Ethics Statement

This study was approved by the institutional review board of the Kunming Institute of Zoology. Written informed consents conforming to the tenets of the Declaration of Helsinki were obtained from each participant prior to the study.

### Study Population and Tissue Samples

Cancerous tissues were collected from 305 Han Chinese with CRC. These patients underwent surgery at the First Affiliated Hospital of Kunming Medical University from 2010 to 2011. All patients were histopathogically confirmed to be CRC and received no medical treatment (except for 12 patients) prior to the surgery to remove the tumor. The stage of cancer was classified according to the 7th edition of AJCC Cancer Staging Handbook [21]. Blood samples were obtained from 342 healthy individuals (including 133 reported in our previous study [20]). Demographic information and histopathological characteristics of CRC patients and healthy controls were shown in Table 1. We also collected paracancerous normal tissues for some patients; this normal tissue was located in a region five centimeters away form the boundary of cancerous tissue, and pathological examination of this tissue showed no sign of malignant cells.

**Table 1:** Demographic information and histopathological characteristics of Han Chinese patients with and without colorectal cancer.

	Case^b^	Control
Characteristics	No. (%)	No. (%)
Gender		
Male	155 (50.8)	182 (53.2)
Female	150 (49.2)	160 (46.8)
Age		
≤50 years old	50 (16.4)	227 (66.4)
>50 years old	255 (83.6)	115 (33.6)
Tumor location		
colon	136 (45.3)	–
Rectum	162 (54.0)	–
Colon and Rectum	2 (0.7)	–
Differentiation		
Good	27 (9.0)	–
Moderate	234 (78.0)	–
Poor	35 (11.7)	–
ND	4 (1.3)	–
T stage^a^		
T0	2 (0.7)	–
T1	3 (1.0)	–
T2	49 (16.3)	–
T3	83 (27.7)	–
T4	163 (54.3)	–
N stage^a^		
N0	190 (63.3)	–
N1	69 (23.0)	–
N2	41 (13.7)	–
M stage^a^		
M0	258 (86.0)	–
M1	42 (14.0)	–

a The stage of cancer was classified following the 7th edition of AJCC Cancer Staging Handbook [21].

b Among all 305 cases, five cases lacked histopathological data.

### Genotyping the Promoter Genetic Variants of the CASP8 Gene

Genomic DNA was extracted from cancerous tissues of CRC patients and blood samples of healthy controls by using standard phenol/chloroform method. We followed the same approach and condition described in our previous study to genotype the three genetic variants in the *CASP8* promoter region [20]. In brief, the 6 bp/del and 8 bp/del polymorphisms were determined by polymerase chain reaction (PCR) and polyacrylamide gel electrophoresis (PAGE). SNP rs3769821 was genotyped by PCR-RFLP assay.

### Quantitative RT–PCR for CASP8 mRNA Expression

Total RNA was isolated from paired cancerous and paracancerous normal tissues of 99 CRC patients who received no radiotherapy and/or chemotherapy treatment before the surgery by using TRIZOL (Invitrogen, Carlsbad, CA) according to manufacturer’s instruction. One microgram of total RNA was used to synthesize single-strand cDNA using an oligo (dT) 18-mer as primer and MMLV Reverse Transcriptase (Promega, Madison, WI) in a final reaction volume of 25 µL. Primers CASP8-F (5′-GCAGAGGGAACCTGGTACAT-3′) and CASP8-R (5′-TCATCCTTGTTGCTTACTTCATAG-3′) were used to detect *CASP8* mRNA transcripts A (GenBank accession number NM_001228.4), B (NM_033355.3), C (NM_033356.3), F (NM_001080124.1) and G (NM_001080125.1). Real-time PCR was performed on the IQ2 Real-Time PCR system (Bio-Rad, Hercules, CA) with the SYBR *Premix Ex Taq* II (Tli RNaseH Plus; TaKaRa, Otsu, Shiga) and the following amplification condition: an initial denaturation at 94°C for 3 min, followed by 35 cycles of 94°C for 15 s, 50°C for 15 s, and 72°C for 20 s, and a final extension cycle at 72°C for 5 min. The *GAPDH* (Glyceraldehyde 3-phosphate dehydrogenase) gene was amplified for normalization. We used primer pair 5′- CAACTACATGGTTTACATGTTC -3′/5′-GCCAGTGGACTCCACGAC-3′ and same thermal cycling parameters (except for a change of annealing temperature to 55°C) as that of the *CASP8* gene to amplify the *GAPDH* gene. Each sample was performed in two duplicates.

### Western Blot Analysis for CASP8 Protein

Tissues were washed with cold ACK buffer to eliminate red blood cells and were mashed in lysis buffer supplied with protease inhibitors by using the Pellet Pestle (Sigma-Aldrich, St. Louis, MO). Protein concentrations were determined by the BCA assay according to the manufacturer’s instruction (Beyotime, Haimen, Jiangsu) using bovine serum albumin as a standard. Twenty-five micrograms of total protein were separated on 15% SDS-PAGE and transferred to a PVDF membrane (Roche Diagnosis, Indianapolis, IN). After blocking with 5% non-fat milk for 2 h at room temperature, membrane was blotted with mouse anti-caspase-8 antibody (1∶4000, Cell Signaling Technology, Danvers, MA) at 4°C overnight. Membrane was washed with TBST three times for 10 min each, followed by incubation with goat anti-mouse IgG secondary antibody (1∶10000, KPL, Gaithersburg, MD) for 1 h at room temperature. Membrane was washed with TBST as described above and developed using the Immobilon Western Chemiluminescent HRP substrate (Millipore, Billerica, MA). The β-actin was quantified in each sample following same procedure using mouse anti-β-actin antibody (1∶100000, Zhongshan Goden Bridge Biotechnology Co., Ltd, Beijing) for normalization. The density of each protein band was calculated using the Image J software (NIH, Bethesda, MD).

### Statistical Analysis

Statistical analysis was performed using the R program (Version 2.11.1, Vienna, Austria) and a *P* value less than 0.05 was considered as statistically significant. Power calculations were performed by using the Quanto software [22]. Deviation from the Hardy–Weinberg equilibrium (HWE) was assessed for each variant by using the *χ* ^2^-test (df = 1). Allele frequencies of the three variants between cases and controls were also compared by using the *χ* ^2^-test. The pairwise D’ value across variants rs3834129, rs3769821 and rs113686495 in cases and controls were determined by Haploview v4.2 [23]. Haplotypes and their frequencies were estimated based on the Bayesian method by using Phase 2.1 [24]. Unconditional logistic regression analysis was employed to calculate the odds ratio (OR) and 95% confidence intervals (CI), for estimating potential association of different genotypes of rs3834129, rs3769821, and rs113686495 with CRC. Genotypes 6 bp/6 bp of rs3834129, TT of rs3769821, and del/del of rs113686495 and the main haplotype were used as the reference adjusted for age (≤50 and >50 years old) and gender. Paired *t*-test was used to determine the difference of the *CASP8* gene expression levels between two groups. ANOVA was used to compare the mean level of the *CASP8* gene expression among groups more than two.

## Results

### Lack of Association between Three Genetic Variants of the CASP8 Promoter and CRC

Our sample size (305 patients versus 342 controls) under matched case-control design with a log-additive inheritance mode had sufficient statistical power for the association study. Among the three variants analyzed in the control population, minor allele frequency (MAF) ranged from 21.1% to 26.7%. Considering MAF of 0.211, the statistical power to detect an odds ratio (OR) value of 1.5 for risk allele was expected to be 85%, whereas the power for MAF of 0.267 was expected to be 90%.

The allele frequencies of rs3834129, rs3769821, and rs113686495 of the *CASP8* gene promoter in case and control groups were listed in Table 2. Genotype distribution of all these variants was not deviated from HWE. No statistically significant difference was observed between the cases and controls for each allele of rs3834129, rs3769821, and rs113686495. Note that there were some slight differences between the allele frequencies of rs3834129 in our samples and the CRC samples from Sun et al. [14] (Table 2), which might reflect regional difference. There was no association of certain genotype of the three variants with CRC (Table 3).

**Table 2:** Allele frequencies of rs3834129, rs3769821 and rs113686495 in Han Chinese patients with and without colorectal cancer.

Sample	N	rs3834129		*P* *	rs3769821		*P* *	rs113686495		*P* *
		6 bp, n (%)	del, n (%)		T, n (%)	C, n (%)		del, n (%)	8 bp, n (%)	
Case^a^	305	481 (78.85)	129 (21.15)	0.98	436 (71.48)	174 (28.72)	0.52	446 (73.11)	164 (26.89)	0.60
Control^b^	342	539 (78.80)	145 (21.20)		501 (73.25)	183 (26.75)		510 (74.56)	174 (25.44)	
Case^c^	918	1,490 (81.15)	346 (18.85)	–	–	–	–	–	–	–
Control^c^	890	1,360 (76.40)	420 (23.60)		–	–		–	–	

a One patient failed to be genotyped and was excluded.

b Including 133 control samples that were reported in Xiao et al. [20].

c Cases and controls were taken from Sun et al. [14].

* Two-sided Chi-square test with Yate’s correction.

**Table 3:** Genotypes of the three *CASP8* gene promoter variants in Han Chinese with and without colorectal cancer.

Genotype	rs3834129				Genotype	rs3769821				Genotype	rs113686495			
	Cases, n (%)	Controls, n (%)	OR (95% CI)	*P* *		Cases,n (%)	Controls,n (%)	OR (95% CI)	*P* *		Cases,n (%)	Controls,n (%)	OR (95% CI)	*P* *
	n = 305	n = 342				n = 305	n = 342				n = 305	n = 342		
6 bp/6 bp	187 (61.31)	212 (61.99)	reference		TT	159 (52.13)	180 (52.63)	reference		del/del	162 (53.11)	187 (54.68)	reference	
6 bp/del	107 (35.08)	115(33.63)	1.14 (0.78–1.68)	0.49	TC	118 (38.69)	141 (41.23)	0.90 (0.62–1.31)	0.58	del/8 bp	122 (40.00)	136 (39.77)	0.96 (0.66–1.40)	0.83
del/del	11 (3.61)	15 (4.39)	0.88 (0.34–2.23)	0.79	CC	28 (9.18)	21 (6.14)	1.74 (0.86–3.57)	0.13	8 bp/8 bp	21 (6.89)	19 (5.56)	1.23 (0.58–2.66)	0.59
∼/del^a^	118 (38.69)	130 (38.01)	1.11 (0.77–1.61)	0.57	∼/C^b^	146 (47.86)	162 (47.37)	0.99 (0.70–1.43)	0.99	∼/del^c^	143 (46.89)	155 (45.32)	0.99 (0.69–1.42)	0.96

a Including genotypes 6 bp/del and del/del.

b Including genotypes TC and CC.

c Including genotypes del/8 bp and 8 bp/8 bp.

* Unconditional logistic regression analysis adjusted for gender and age (≤50 and >50 years old).

**Figure 1:**
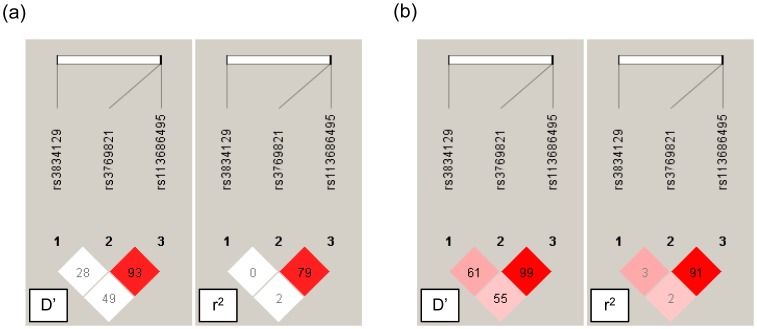
Graphical representation of pairwise D’ and r^2^ values across three genetic variants in the *CASP8* promoter in patients with colorectal cancer (a) and healthy individuals (b). The numbers within the squares displayed for the D’ and r^2^ scores (x100) for pairwise linkage disequilibrium (LD) with a red-to-white gradient reflecting higher to lower LD scores.

Since variants rs3769821 and rs113686495 were in strong linkage disequilibrium in both case and control populations (Figure 1), we inferred haplotypes based on variants rs3834129 and rs3769821 only and assessed potential association between haplotype and CRC risk. Similarly, we observed no association of haplotype with CRC (Table 4). Taken together, our results suggested that genetic variants in the *CASP8* gene promoter region did not likely to confer major risk to CRC in Han Chinese from southwest China.

**Table 4:** Haplotypes of the *CASP8* gene promoter variants in Han Chinese with colorectal cancer and healthy individuals.

Haplotype^a^	Case,n (%)	Control,n (%)	OR(95%, CI)	*P* *
H1	318 (55.54%)	363 (54.84%)	reference	
H2	163 (23.31%)	176 (23.96%)	1.05 (0.77–1.42)	0.77
H3+ H4	118+11 (21.14%)	138+7 (21.19%)	1.07 (0.78–1.49)	0.67

a Order of SNP: s3834129-rs3769821; H1: 6 bp-T, H2: 6 bp-C, H3: del-T, H4: del-C.

* Unconditional logistic regression analysis adjusted for gender and age (≤50 and >50 years old).

### CASP8 mRNA Expression Levels in CRC Tissues and the Corresponding Normal Tissues are Similar

**Figure 2:**
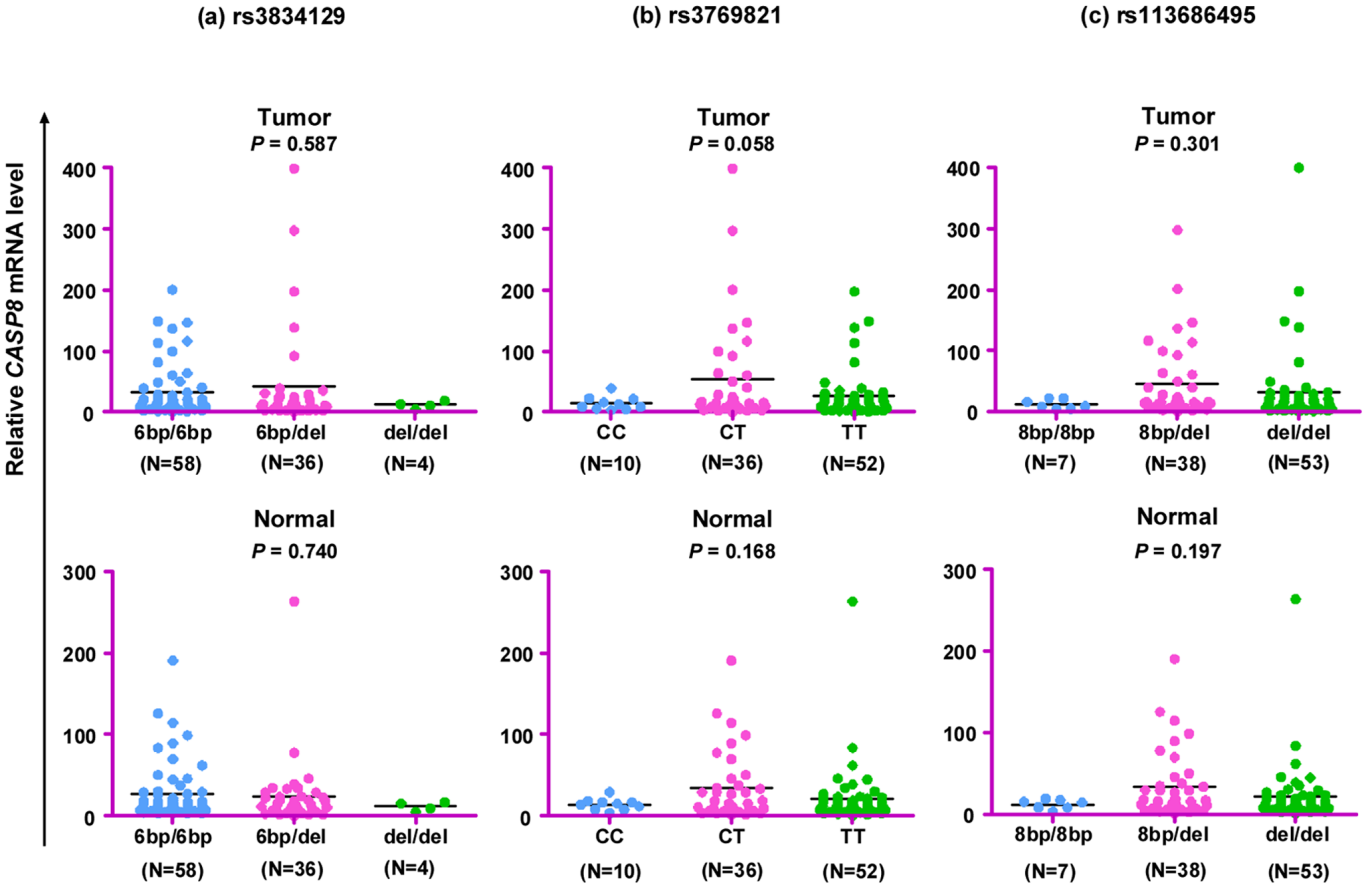
Relative *CASP8* mRNA expression in cancerous and paired paracancerous normal tissues from patients with different genotypes of variants rs3834129 (a), rs3769821 (b), and rs113686495 (c). Among 99 untreated patients measured for the *CASP8* mRNA expression, one patient failed to be genotyped and was excluded.

To examine whether *CASP8* expression levels differ between patients and within matched normal and tumor samples and then to address whether there is any correlation with genotype, we analyzed the *CASP8* mRNA expression level in paired cancerous and paracancerous normal tissues from 99 patients who received no treatment prior to surgery. Similar to genotyping result, there was no statistically significant difference of the *CASP8* mRNA level in either cancerous or paracancerous normal tissues in patients with different genotypes (Figure 2). Note that mRNA expression in tissues with genotypes del/del of rs3834129, CC of rs3769821, and 8 bp/8 bp of rs113686495 showed a relatively lower value than those of the other genotypes (Figure 2), and this might be attributed to smaller number of patients with these genotypes if were not caused by the cis-regulation since these SNPs are in the promoter region of the *CASP8* gene.

**Figure 3:**
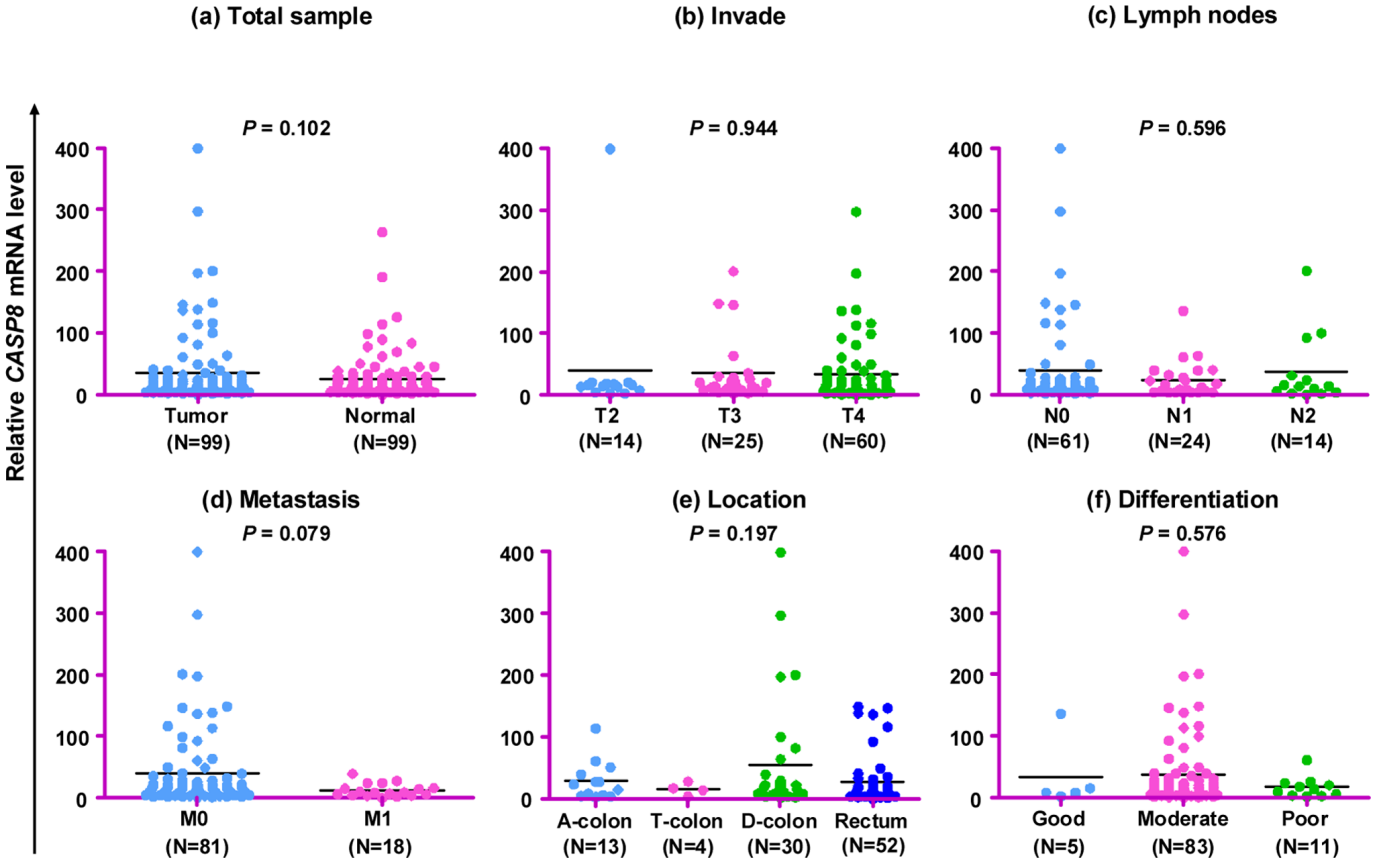
Relative *CASP8* mRNA levels in cancerous and paracancerous normal tissues from patients with different pathological characteristics. The TNM staging was classified according to the 7th edition of AJCC Cancer Staging Handbook [21], and the location and differentiation of cancerous tissues were determined by surgical and histopathological examination, respectively. There was no significant difference of the *CASP8* mRNA expression in cancerous and paracancerous normal tissues from all 99 patients (a). The *CASP8* mRNA expression in cancerous tissues from patients with different clinical characteristics showed no significant difference (b–f). A-colon, T-colon, D-colon and Rectum stand for ascending colon, transverse colon, descending colon and rectum, respectively.

**Figure 4:**
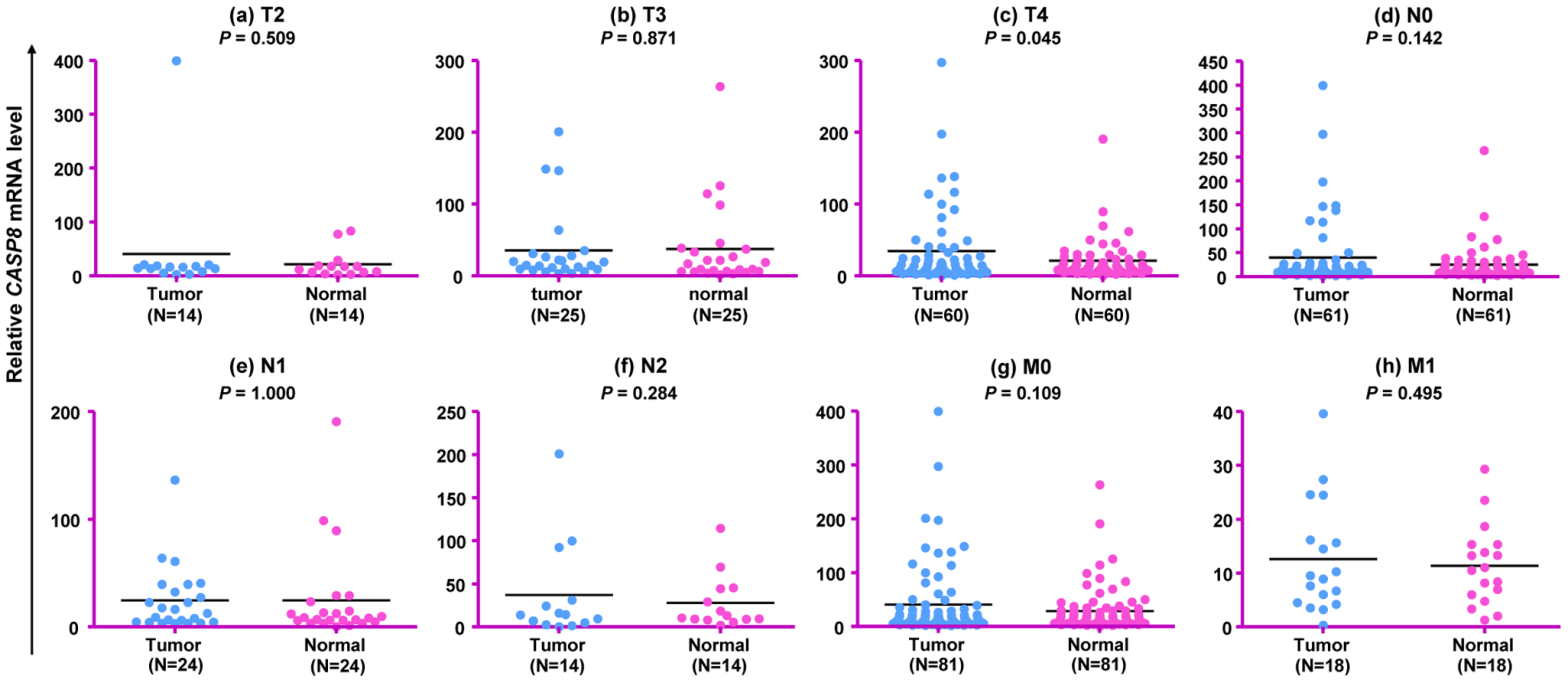
Relative *CASP8* mRNA expression in cancerous and paracancerous normal tissues from patients at different cancer stages. The TNM staging was classified according to the 7th edition of AJCC Cancer Staging Handbook [21].

To detect potential effect of the *CASP8* gene on the pathogenesis and clinical characteristics of CRC, we compared the *CASP8* mRNA expression levels in cancerous tissues and paracancerous normal tissues in all patients but observed no significant difference (*P* = 0.102; Figure 3a). Similarly, the *CASP8* mRNA expression in cancerous tissues from patients with different clinical characteristics showed no significant difference (Figures 3b, c, d, e, f). Cancerous and paracancerous normal tissues from patients at different stages of cancer development and progression had a similar level of *CASP8* mRNA expression (Figure 4), although cancerous tissues from patients at T4 stage had a marginally significant higher mRNA expression than paired paracancerous normal tissues (*P* = 0.045; Figure 4c). Apparently, the *CASP8* gene mRNA expression level was not tightly associated with CRC in our patients.

### CASP8 Protein Level was Significantly Decreased in Cancerous Tissues Comparing with Paired Paracancerous Normal Tissues

**Figure 5:**
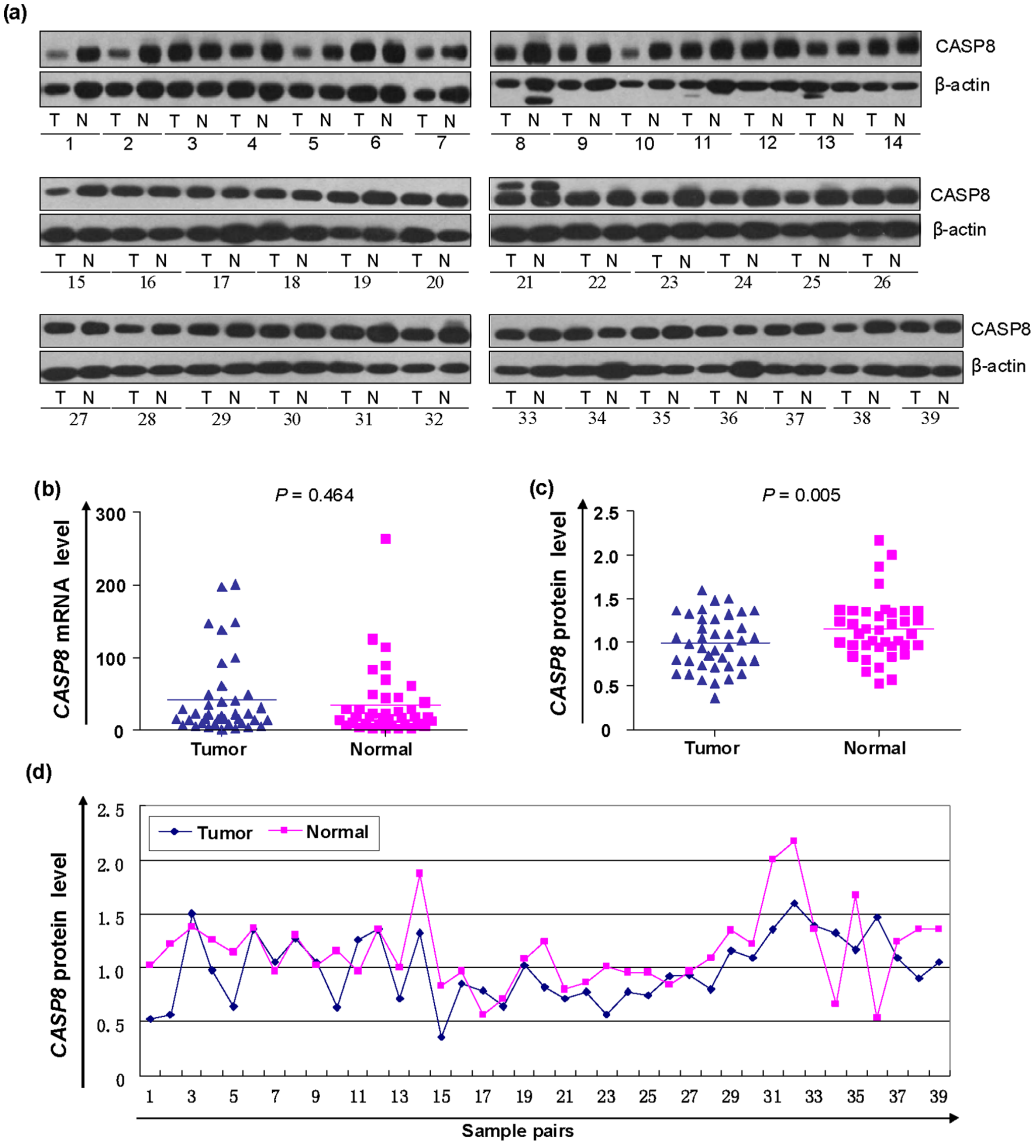
Incoherent expression pattern of the *CASP8* mRNA and protein in paired cancerous tissue (T) and paracancerous normal tissue (N) from 39 patients. The genotype and clinical information for these patients were listed in Table S1.

Many genes were regulated via a post-transcriptional mechanism, e.g. upregulation of placental growth factor by vascular endothelial growth factor in endothelial cells [25]. To investigate whether the *CASP8* gene has a role in CRC development through a post-transcriptional regulation, we selected paired cancerous and paracancerous normal tissues from 39 patients with different *CASP8* promoter genotypes, mRNA expression and quantified protein expression level (Table S1). We observed a significant lower level of CASP8 protein in cancerous tissues than paracancerous tissues (*P* = 0.005), although the *CASP8* mRNA levels in these samples were similar (*P* = 0.464, Figure 5).

## Discussion

Caspase 8 (*CASP8*) is a well-known procurer of death signal in the apoptotic pathway that was involved in death receptor stimulation, permeabilisation of the outer mitochondrial membrane and release of pro-apoptotic proteins into the cytosol from mitochondria [26]. It acts as a crucial defensive barrier against malignant proliferation and tumorigenesis [7], [10], [27]. Previous studies have presented conflicting results regarding the potential role of genetic variants in the *CASP8* gene promoter region in tumorigenesis [14], [15], [16], [17], [18], [20], [28], [29], [30]. In a pioneer study, genetic variant (rs3834129) in the *CASP8* promoter region was associated with a wide range of solid cancers including lung, esophageal, gastric, colorectal, cervical, and breast cancers in Chinese populations by affecting the Sp1 transcriptional factor binding site and the *CASP8* gene expression [14]. This initial finding was verified in subsequent case-control association studies in independent Chinese samples with bladder cancer [16] and coal workers’ pneumoconiosis [15]. However, several other reports failed to replicate the association between this 6 bp/del polymorphism and multiple cancers in different populations [18], [30], [31], [32]. These inconsistent results might be explained by different genetic backgrounds of populations in different studies [33], as the frequency of 6-bp del allele was significantly different between Asian and Caucasian populations (22.4% vs. 49.1%) [32], [34]. In a recent study, Lan and coworkers [19] provided evidence that another SNP rs3769821 in the *CASP8* promoter region was significantly associated with genetic risk of NHL. However, we failed to replicate this result in Han Chinese with NHL and cellular luciferase assay showed no difference of the promoter region containing different alleles [20]. Hitherto, whether the *CASP8* gene expression could affect the pathogenesis of CRC is still controversial [35], [36], [37]. There is a necessity to reappraise the potential effect of promoter variants and expression of the *CASP8* gene on CRC.

In this study, by analyzing CRC patients from Kunming, Yunnan Province, we intended to answer whether the *CASP8* gene was actively involved in the development of CRC. We first screened three genetic variants in the *CASP8* gene promoter region, in which two have been reported to be associated with solid tumors (rs3834129; [14]) and NHL (rs3769821; [19]). We then quantified the *CASP8* mRNA level in paired cancerous and paracancerous normal tissues to further discern potential correlation between the *CASP8* genotypes and mRNA expression. We found no association of the *CASP8* promoter variants with CRC in the case and control samples (Tables 2 and 3). Moreover, we did not find any statistically significant difference of the *CASP8* mRNA level in patients with different genotypes (Figure 2). These negative results were consistent with our previous luciferase assay [20], in which we observed no difference for vectors with different alleles in the *CASP8* promoter.

The available clinical data for patients offered us an opportunity to evaluate the correlation between the *CASP8* gene expression and development of CRC. By grouping patients according tumor stage, metastasis, and differentiation status, we found no significant difference of the *CASP8* mRNA expression levels between cancerous and paracancerous normal tissues and between patients with different clinical features (Figures 3 and 4), suggesting that the *CASP8* mRNA expression might not play a crucial role in CRC. Similar to our finding, Pan et al. [36] also did not identify any significant difference of the *CASP8* mRNA levels in normal mucosa, polyps, and CRC. However, in contrary to two previously reported studies that the *CASP8* expression was upregulated during colorectal carcinogenesis [35], [37], we showed that the CASP8 protein level in cancerous tissues was significantly lower than that of paired paracancerous normal tissues (Figure 5). The incoherent pattern of the *CASP8* mRNA and protein expression levels in this study suggested that a post-transcriptional regulation, such as excessive ubiquitin of CASP8 protein in tumor tissue and/or miRNA regulation, may play a crucial role in the development of CRC. Further characterization should be performed in the future to solidify this speculation.

Our study had several limitations. First, the sample size (305 cases and 342 controls) for the association analysis was relatively small and only three variants in the promoter region of the *CASP8* gene were genotyped, which might not have sufficient power to detect the effects assuming a relatively low odds ratio. Further studies with larger number of samples and more genetic variants will be essential to verify our conclusion. Second, we only analyzed *CASP8* mRNA and protein expression in a subset of CRC patients. As mentioned above, we observed a reduced expression within certain genotypes (Figure 2). More patients should be analyzed to definitely exclude potential bias caused by small numbers of patients in these groups. Third, we did not pick up all *CASP8* transcripts/precursors in this study. Our measurement for *CASP8* mRNA transcripts A, B, C, G and G in one assay could not distinguish which transcript plays an important role in the tissue of interest. Finally, we did not screen mutation in the coding region of the *CASP8* gene. It remains unknown whether specific CASP8 mutant would affect CRC development. Kim and coworkers [38] reported that the presence of mutations in the *CASP8* gene coding region could cause dysfunction of the apoptotic pathway, which may finally contribute to the development of CRC.

Collectively, we speculated that the *CASP8* gene might initiate CRC development and progression, if any, via regulation of protein level and/or coding region functional mutation(s), instead of mRNA expression.

### Conclusions

We found that genetic variants rs3834129, rs3769821, and rs113686495 in the *CASP8* promoter region were not associated with genetic susceptibility to CRC in Han Chinese from Southwest China. Further analyses of the *CASP8* gene expression in cancerous and paracancerous tissues showed no correlation of mRNA expression level with different genotypes and progress of CRC. However, we found that CASP8 protein was significant lower in cancerous tissues than in paired paracancerous normal tissues, albeit both samples had similar level of mRNA expression. These results suggested that aberrant expression and/or malfunction of the CASP8 protein would play an active role in CRC development and progression. Independent population with larger sample size and functional assays are needed to further confirm our findings.

## Supporting Information

Table S1 **Genotype and pathological information for 39 patients that were analyzed for CASP8 protein expression.**
(DOC)
