# Supplementary material for: Genetic Polymorphisms of the CASP8 Gene Promoter May Not Be Associated with Colorectal Cancer in Han Chinese from Southwest China
Source: PLoS One. 2013 Jul 2;8(7):e67577. doi: 10.1371/journal.pone.0067577 (PMC3699664; doi:10.1371/journal.pone.0067577)
Supplement: Table S1 — Genotype and pathological information for 39 patients that were analyzed for CASP8 protein expression. (DOC) [file pone.0067577.s001.doc]

**Table S1.** Genotype and pathological information for 39 patients that were analyzed for CASP8 protein expression

| Sample  No. | Patient ID | Genotype | | | Tumor location | Clinical features | |
| --- | --- | --- | --- | --- | --- | --- | --- |
|  |  | rs3834129 | rs3769821 | rs113686495 |  | Differentiation | TNM ^a^ |
| 1 | 5 | 6bp/del | C/C | 8bp/8bp | Rectum | Moderate | T2N0M0 |
| 2 | 18 | 6bp/6bp | C/C | 8bp/8bp | Rectum | Moderate | T4N0M0 |
| 3 | 22 | 6bp/6bp | T/C | 8bp/del | Descending colon | Moderate | T4N1M1 |
| 4 | 29 | 6bp/del | T/T | del/del | Descending colon | Moderate | T4N0M0 |
| 5 | 42 | 6bp/6bp | T/T | del/del | Rectum | Moderate | T3N0M0 |
| 6 | 48 | del/del | C/C | 8bp/del | Rectum | Moderate | T3N0M0 |
| 7 | 51 | 6bp/6bp | T/C | 8bp/del | Rectum | Good | T4N0M1 |
| 8 | 62 | 6bp/del | T/T | del/del | Rectum | Moderate | T3N0M0 |
| 9 | 63 | del/del | T/T | del/del | Rectum | Moderate | T4N2M0 |
| 10 | 84 | 6bp/6bp | T/C | 8bp/del | Ascending colon | Moderate | T4N0M0 |
| 11 | 100 | 6bp/6bp | T/C | 8bp/del | Rectum | Moderate | T3N0M0 |
| 12 | 102 | 6bp/6bp | T/C | 8bp/del | Descending colon | Moderate | T3N2M0 |
| 13 | 107 | 6bp/6bp | T/T | del/del | Rectum | Moderate | T4N0M0 |
| 14 | 111 | 6bp/del | C/C | 8bp/8bp | Ascending colon | Moderate | T4N1M0 |
| 15 | 32 | 6bp/del | T/T | del/del | Descending colon | Moderate | T3N0M0 |
| 16 | 33 | 6bp/del | T/T | del/del | Rectum | Moderate | T2N0M0 |
| 17 | 35 | 6bp/6bp | T/T | 8bp/del | Rectum | Moderate | T4N1M0 |
| 18 | 36 | 6bp/del | T/C | 8bp/del | Rectum | Moderate | T4N2M1 |
| 19 | 37 | 6bp/6bp | C/C | 8bp/del | Ascending colon | Moderate | T4N1M1 |
| 20 | 38 | 6bp/6bp | T/T | del/del | Rectum | poor | T4N2M0 |
| 21 | 39 | 6bp/6bp | T/T | del/del | Descending colon | Moderate | T4N2M0 |
| 22 | 40 | 6bp/del | T/T | del/del | Rectum | Moderate | T4N0M0 |
| 23 | 41 | 6bp/6bp | T/C | 8bp/del | Ascending colon | poor | T4N1M0 |
| 24 | 43 | 6bp/6bp | T/C | 8bp/del | Descending colon | Moderate | T4N2M0 |
| 25 | 44 | 6bp/del | T/T | del/del | Descending colon | Moderate | T4N0M0 |
| 26 | 45 | 6bp/6bp | T/T | del/del | Descending colon | poor | T3N0M0 |
| 27 | 52 | 6bp/del | T/C | 8bp/del | Rectum | Moderate | T4N2M0 |
| 28 | 53 | 6bp/del | T/T | del/del | Rectum | Moderate | T4N0M0 |
| 29 | 54 | 6bp/6bp | T/T | del/del | Rectum | Moderate | T4N1M0 |
| 30 | 55 | 6bp/6bp | T/C | 8bp/del | Rectum | Moderate | T4N1M0 |
| 31 | 58 | 6bp/del | T/T | del/del | Rectum | Moderate | T4N0M0 |
| 32 | 59 | 6bp/del | C/C | 8bp/8bp | Rectum | Moderate | T4N2M1 |
| 33 | 72 | 6bp/6bp | T/C | 8bp/del | Descending colon | Moderate | T3N0M0 |
| 34 | 67 | 6bp/del | T/T | del/del | Descending colon | Moderate | T3N0M0 |
| 35 | 68 | 6bp/6bp | T/T | del/del | Rectum | Moderate | T4N2M0 |
| 36 | 69 | 6bp/6bp | T/T | del/del | Rectum | Moderate | T4N0M0 |
| 37 | 70 | 6bp/6bp | T/T | del/del | Rectum | Moderate | T2N0M0 |
| 38 | 71 | 6bp/del | T/T | del/del | Rectum | Moderate | T3N2M0 |
| 39 | 65 | 6bp/6bp | T/C | 8bp/del | Descending colon | Moderate | T4N1M1 |

^a^ The stage of cancer was classified following the 7th edition of AJCC Cancer Staging Handbook [21].
